# Fusing literature and full network data improves disease similarity computation

**DOI:** 10.1186/s12859-016-1205-4

**Published:** 2016-08-30

**Authors:** Ping Li, Yaling Nie, Jingkai Yu

**Affiliations:** 1 State Key Laboratory of Biochemical Engineering, Institute of Process Engineering, Chinese Academy of Sciences, Beijing, 100190 China; 2 University of Chinese Academy of Sciences, Beijing, 100049 China

**Keywords:** Disease similarity, MedSim, NetSim, MedNetSim, Random walk with Restart

## Abstract

**Background:**

Identifying relatedness among diseases could help deepen understanding for the underlying pathogenic mechanisms of diseases, and facilitate drug repositioning projects. A number of methods for computing disease similarity had been developed; however, none of them were designed to utilize information of the entire protein interaction network, using instead only those interactions involving disease causing genes. Most of previously published methods required gene-disease association data, unfortunately, many diseases still have very few or no associated genes, which impeded broad adoption of those methods. In this study, we propose a new method (MedNetSim) for computing disease similarity by integrating medical literature and protein interaction network. MedNetSim consists of a network-based method (NetSim), which employs the entire protein interaction network, and a MEDLINE-based method (MedSim), which computes disease similarity by mining the biomedical literature.

**Results:**

Among function-based methods, NetSim achieved the best performance. Its average AUC (area under the receiver operating characteristic curve) reached 95.2 %. MedSim, whose performance was even comparable to some function-based methods, acquired the highest average AUC in all semantic-based methods. Integration of MedSim and NetSim (MedNetSim) further improved the average AUC to 96.4 %. We further studied the effectiveness of different data sources. It was found that quality of protein interaction data was more important than its volume. On the contrary, higher volume of gene-disease association data was more beneficial, even with a lower reliability. Utilizing higher volume of disease-related gene data further improved the average AUC of MedNetSim and NetSim to 97.5 % and 96.7 %, respectively.

**Conclusions:**

Integrating biomedical literature and protein interaction network can be an effective way to compute disease similarity. Lacking sufficient disease-related gene data, literature-based methods such as MedSim can be a great addition to function-based algorithms. It may be beneficial to steer more resources torward studying gene-disease associations and improving the quality of protein interaction data. Disease similarities can be computed using the proposed methods at http://www.digintelli.com:8000/.

**Electronic supplementary material:**

The online version of this article (doi:10.1186/s12859-016-1205-4) contains supplementary material, which is available to authorized users.

## Background

Discovering closely related diseases could be helpful in revealing their common pathophysiology [1, 2]. It may also be useful for identifying novel drug indications [3], as similar diseases may have the same or similar therapeutic targets, which suggests they could be treated with the same or similar drugs. There has been a growing interest in quantitatively measuring similarities between diseases [4–7].

Phenotypic similarity plays an important role in a number of biological and biomedical applications [8]. During the past years, based on the Human Phenotype Ontology (HPO) [9], researchers had designed several methods to find related diseases and predict disease-causing genes, such as Phenomizer [10], Exomiser [11] and PhenIX [12]. The HPO provides a controlled and standardized vocabulary of phenotypic abnormalities that characterize human diseases. Phenotype similarity also, becomes the most common way to define classification rules for diseases. The classification of disease terms in Medical Subject Headings (MeSH) [13] and Disease Ontology (DO) [14] are taking this approach. To quantify disease similarity, several semantic-based methods had thus been proposed based on HPO, MeSH or DO, such as Resnik [15], Lin [16] and Wang [17]. Resnik’s method measures disease similarity based on information content (IC) of the most informative common ancestor (MICA) between two terms. Besides IC of MICA, Lin’s method also considers the IC of the two compared diseases [16]. Wang et al.’s method [17] computes similarity of a disease pair by considering the contribution of all common ancestors in the ontology. It had been successfully applied to compute similarity between MeSH [18] terms. All of those semantic-based methods exploited disease associations based on ontologies and/or gene annotations. They did not, however, consider the functional associations between disease-related gene sets. The BOG (based on overlapping gene sets) method was thus designed by Mathur and Dinakarpandian [19], which calculates disease similarity by exploiting the co-occurrence of disease-related genes. Mathur et al. [20] also devised a process-similarity based (PSB) method. Instead of defining disease similarity as a function of genes, PSB computes disease similarity based on Gene Ontology (GO) [21] biological process terms associated with those genes. PSB achieved a better performance than BOG [20]. Functional associations between genes involve not only GO terms [22], but also co-expression [23], protein-protein interaction [24], etc. Cheng et al. recently presented the method FunSim [25], which measures disease similarity using a weighted human protein interaction network. The first neighbors of disease-related genes in the protein network were taken into account. FunSim further improved the results of PSB [25].

Although a number of methods for computing disease similarity had been developed, no method had been proposed to take advantage of the entire protein interaction network, beyond using only the first neighbors. A network-based method (NetSim) is proposed which takes advantage of the entire interaction network. The effectiveness of different data sources were also evaluated, including gene-disease associations and protein-protein interactions. Most of the previously developed methods were based on disease-related genes. However, many diseases still have very few or no associated genes. Relying entirely on disease-related genes greatly limits the utility of those methods. To overcome the limitation, a new semantic-based similarity measure (MedSim) is developed to compute disease similarity based on the MEDLINE database. MedSim and NetSim were eventually integrated into MedNetSim to further improve computing performance.

## Methods

### Diseases and gene-disease association databases

The disease terms in DO were chosen as the vocabulary for describing diseases. DO database is a biomedical resource of disease concepts with stable identifiers organized by disease etiology [14]. It contains 6,457 non-obsolete disease terms and 6,819 ‘IS_A’ relationships among diseases. The non-obsolete disease terms was used as the disease vocabulary. Each disease in DO has a unique identifier, called DOID.

**Fig 1:**
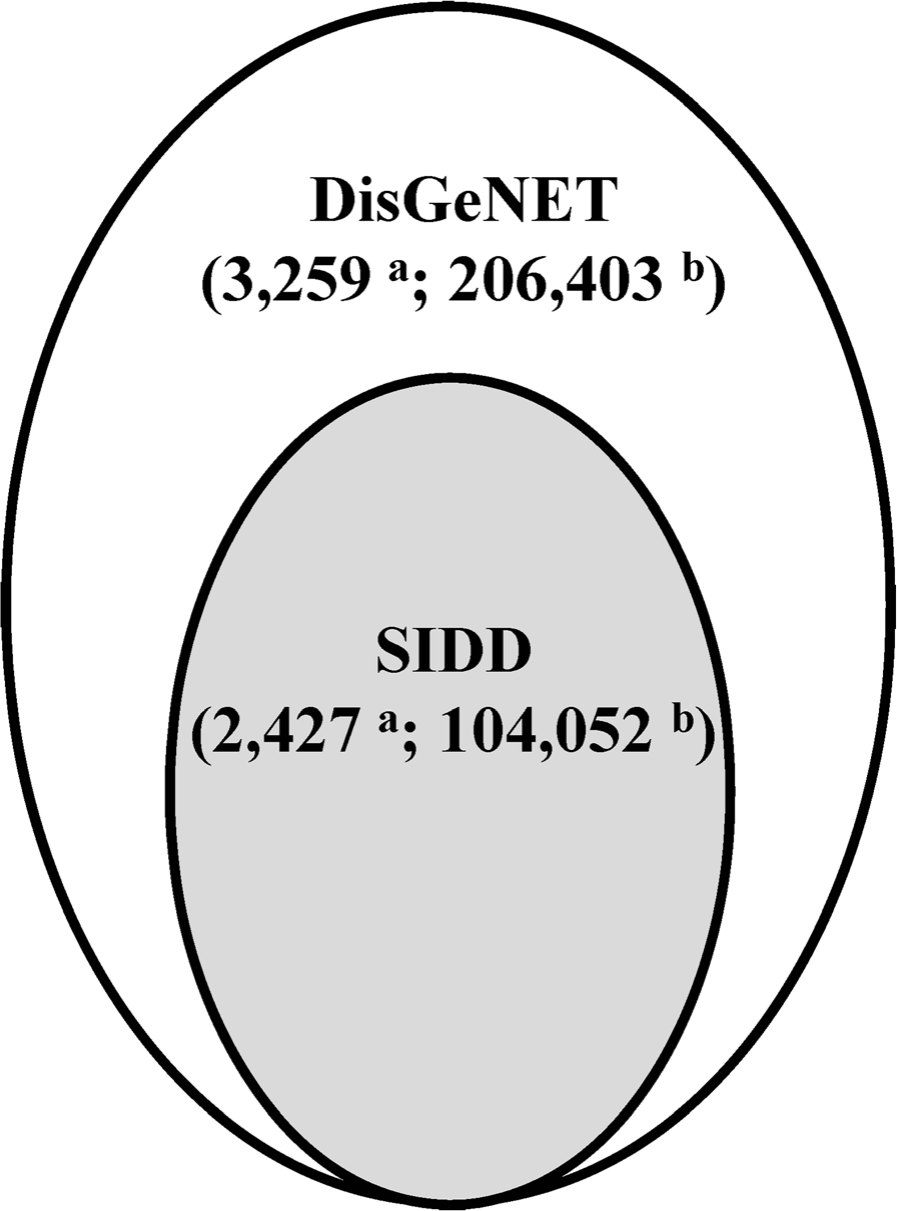
Gene-disease association databases. **a** : The number of diseases, **b**: The number of associations between genes and diseases

SIDD [26] and DisGeNET [27] were adopted as two disease-gene association databases (Fig. 1). SIDD integrated five disease-related gene databases: GeneRIF [28], Online Mendelian Inheritance in Man (OMIM) [29], Comparative Toxicogenomics Database (CTD) [30], Genetic Association Database (GAD) [31], and SpliceDisease [32]. In total, SIDD contains 2,427 diseases and 104,052 gene-disease associations (see Additional file 1). The DisGeNET [27] database integrated human gene-disease associations from various expert curated databases and text-mining derived associations including Mendelian, complex and environmental diseases. Compared to SIDD, DisGeNET had more lower reliability disease-gene associations based on literature mining, i.e., LHGDN [33] and BeFree data [34]. DisGeNET contains 14,619 diseases and 429,111 gene-disease associations. UMLS ID (Unified Medical Language System Identifier) was used as the unique identifier for each disease in DisGeNET. We mapped UMLS ID to DOID, which produced 3,259 disease terms and 206,403 gene-disease associations (see Additional file 2). Almost every disease term in DisGeNET has more associated genes than that in SIDD. All source data were downloaded until April 30, 2015.

### Protein interaction datasets

**Fig 2:**
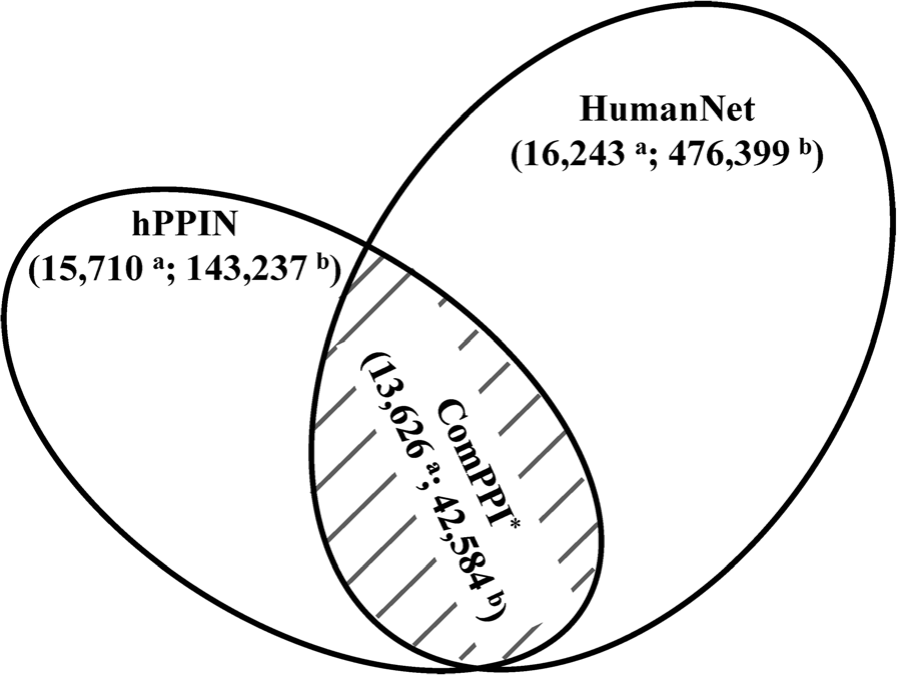
Protein interaction datasets. **a**: The number of genes, **b**: The number of interactions between genes, *: The common protein-protein interactions

Two protein interaction datasets were used (Fig. 2). One is hPPIN, built in house, which integrated four existing protein interaction databases, i.e., BioGrid [35], HPRD [36], IntAct [37], and HomoMINT [38]. Protein identifiers were mapped to the genes coding for the proteins, and redundant interactions were removed. The acquired protein interaction network covered 15,710 human genes and 143,237 interactions (Fig. 2). The other is HumanNet [39], which is a genome-scale functional network for human genes. To build HumanNet, 21 diverse functional genomics and proteomics datasets were evaluated for their tendencies to link human genes in the same biological processes. Pairwise gene linkages derived from the individual datasets were then integrated into a comprehensive HumanNet [39]. HumanNet contains 476,399 functional linkages among 16,243 human genes (Fig. 2). Unlike hPPIN which mainly focuses on experimentally verified protein interactions, HumanNet was constructed based on the functional probability that two genes belonged to the same biological processes. The two protein interaction datasets have 13,626 genes and 42,584 interactions in common (called comPPI, Fig. 2). Additionally, different proportions of hPPIN (5 %, 10 %, 20 %, 40 %, 60 %, 80 %, 90 %) were randomly sampled 20 times and used as the protein interaction datasets to evaulate the impact of data volume on the proposed method.

### Medline-based disease similarity (MedSim)

**Fig 3:**
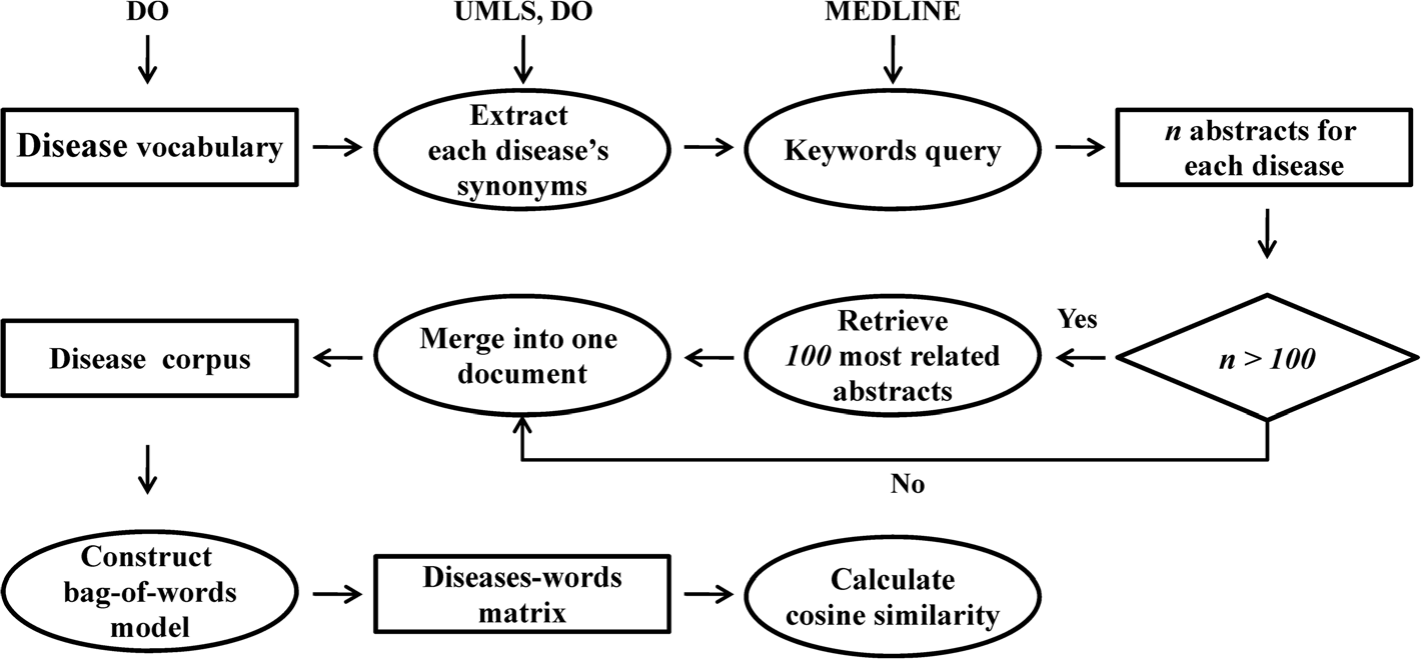
Overview of MedSim. DO: Human Disease Ontology database; UMLS: Unified Medical Language System

Biomedical literature contains rich and diverse information, such as disease symptoms, pathogenesis, therapeutic drugs, and so on. Features representing diseases were generated through mining the biomedical literature corpus; the features were then utilized to compute disease similarity (MedSim method, Fig. 3). MedSim was not limited to use only one aspect of disease information (i.e., disease-related genes), but took advantages of all relevant information that had already been archived in the literature.

#### Disease corpus

The text corpus contains all MEDLINE abstracts published up to year 2015. The non-obsolete disease terms in DO were used as the disease vocabulary. Each disease term was mapped to Unified Medical Language System (UMLS) [40] so that its synonyms could be retrieved. Synonyms were taken directly from DO for diseases that could not be mapped to UMLS. Every disease term and its synonyms were then used as keywords to perform keyword-based queries into MEDLINE to retrieve abstracts related to that disease. To limit computational cost, only the top 100 most relevant abstracts were selected to construct the bag-of-words model for diseases. The relevance of an abstract to a disease was defined in Eq. 1.

1 $$ {R}_{abstract}={\displaystyle \sum_W{W}_{df}\times {W}_{of}} $$

Where *W*_*df*_ and *W*_*of*_ represent document frequency and occurrence frequency of a word X, respectively. Document frequency *W*_*df*_ is the proportion of abstracts that contain word X. *W*_*df*_ represents the relevance of word X to a disease. Occurrence frequency *W*_*of*_ represents the number of times word X occurs in an abstract, measuring the importance of word X in a specific abstract. For a specific disease, *W* is defined as the set of nouns (Xs) which appeared in abstracts when *W*_*df*_ is greater than 0.005. Larger *R*_*abstract*_ means that an abstract is more closely related to the disease. Some diseases were not yet broadly studied, so their number of retrieved abstracts can be less than 100. For those cases, all retrieved abstracts were used. For each disease, the selected most relevant abstracts were merged into one combined document. At the end of preprocessing, every disease was associated with one document. These documents together made up the disease corpus.

#### Constructing the bag-of-words model and computing MedSim

The disease corpus was tokenized to obtain word vocabulary, using Python package NLTK (Nature Language Toolkit, www.nltk.org) to remove non-alphabetic words and reduce inflected/derived words to their stem. Overly common (appeared in more than 60 % of the documents) or rare (appeared in less than 4 documents) words were removed, as those words could not provide meaningful information. Each disease was then represented by a word vector, whose dimensionality is the size of the word vocabulary. Each dimension was assigned a weight (TF-IDF, that is, TF times IDF) based on term frequency (TF) and inverse document frequency (IDF) values. TF is the number of times a word appears in a document. IDF represents the inverse of the number of documents containing the word. TF-IDF assigns larger weights to words that appeared more often in a document but only in a small percentage of all documents, as those words are important and informative for that document. With diseases represented as TF-IDF weighted vectors, the MedSim of two diseases was measured by calculating the cosine similarity of the two vectors. Python package scikit-learn [41] was used to perform the computation.

### Network-based disease similarity (NetSim)

Previously published methods weren’t designed to utilize the entire protein interaction network. They instead focused only on the disease-related genes or their first neighbors in the network. To take full advantage of the entire protein interaction network, random walk with restart (RWR) [42, 43] (see [44] for working details) was used to measure Functional Relevance (FR) between a gene *g* and a gene set *G*, which is described in Eq. 2.

2 $$ F{R}_G(g)=\left\{\begin{array}{l}{P}_{RWR}\kern3.1em g\in protein\kern0.5em  interaction\kern0.5em  network\\ {}1\kern5em g\notin protein\kern0.5em  interaction\kern0.5em  network\kern0.5em  and\kern0.5em g\in G\\ {}0\kern5em g\notin protein\kern0.5em  interaction\kern0.5em  network\kern0.5em  and\kern0.5em g\notin G\end{array}\right) $$

Where gene set *G* was defined to be the seed genes*,* that is, the known set of genes associated with a disease. The initial probability of each seed genes was set to 1.0. *P*_*RWR*_ represents the acquired steady-state probability of gene *g* after running RWR in the whole protein interaction network. A larger probability (*FR*_*G*_*(g)*) will be assigned to gene *g* when it sits more closely to the gene set *G* in the network according to Eq. 2, which means that gene *g* are more functionally related with gene set *G*.

Suppose that *G*_*1*_ = {*g*_*11*_*,g*_*12*_*,…*} and *G*_*2*_ = {*g*_*21*_*,g*_*22*_*,…*} are the seed gene sets for disease *d*_*1*_ and *d*_*2*_, respectively. Then, the NetSim of *d*_*1*_ and *d*_*2*_ is defined in Eq. 3.

3 $$ \begin{array}{l} NeSim\left({G}_1,{G}_2\right)=\frac{{\displaystyle \sum_{1\le i\le len\left({G}_1\right)}F{R}_{G_2}\left({g}_{1i}\right)+}{\displaystyle \sum_{1\le j\le len\left({G}_2\right)}F{R}_{G_1}\left({g}_{2j}\right)}}{len\left({G}_1\right)+len\left({G}_2\right)},\\ {}{g}_{1i}\in {G}_1,{g}_{2j}\in {G}_2\end{array} $$

Where *len(G*_*1*_*)* and *len(G*_*2*_*)* are the number of genes in *G*_*1*_ and *G*_*2*_, respectively. The numerator is the sum of functional relevance of *g*_*1i*_ to *G*_*2*_ and *g*_*2j*_ to *G*_*1*_. A higher NetSim value represents closer connection between *G*_*1*_ and *G*_*2*_, which suggests closer ties between diseases *d*_*1*_ and *d*_*2*_.

MedSim and NetSim is combined into MedNetSim, which is defined in Eq. 4.

4 $$ MedNetSim\left({d}_1,{d}_2\right)= MedSim\left({d}_1,{d}_2\right)\times NetSim\left({G}_1,{G}_2\right) $$

Where *d*_*1*_ and *d*_*2*_ are two diseases in DO, *G*_*1*_ and *G*_*2*_ are the seed gene sets for *d*_*1*_ and *d*_*2*_, respectively.

### Performance evaluation

Similarities of disease pairs in the benchmark set and the random set were calculated and ranked in descending order, receiver operating characteristic (ROC) [45] curves were then drawn to evaluate and quantify the predictive power of the proposed methods. A ROC curve is a plot of the true positive rate of a classifier as a function of the false positive rate. The area under the ROC curve (AUC) is used as a quantitative measure of a classifier’s quality [46]. Disease pairs in the benchmark set and the random set are defined as positives and negatives, respectively. True positives are the disease pairs in the benchmark set that are correctly predicted by a classifier, and false positives are those disease pairs from the random set that are predicted to be positives but not found in the benchmark set. More percentage of disease pairs in the benchmark set receiving higher rankings means better AUC values. The benchmark set was taken from reference [25]. It had 47 diseases and 70 disease pairs (see Additional file 3) with high similarity derived from two manually checked datasets by Suthram et al. [2] and Pakhomov et al. [47]. Cancers were omitted. The benchmark set contains disease pairs that are expected to be related to each other, such as Alzheimer’s disease (DOID: 10652) and schizophrenia (DOID: 5419), diabetes mellitus (DOID: 9351) and obesity (DOID: 9970). It also includes some pairs that are not apparently related, but were found to be correlated by various evidences, such as asthma (DOID: 2841) and diabetes mellitus, malaria (DOID: 12365) and anemia (DOID: 2355). 700 disease pairs were randomly selected from DO to generate a random set, with disease pairs from the benchmark set removed from the generated random set. To get an average AUC of the proposed methods, the above experiment was iterated 50 times by calculating similarities of disease pairs in the benchmark set and 50 random sets.

MedSim was compared with other semantic-based methods including Resnik [15], Lin [16] and Wang [17], based on HPO and DO, respectively. For each disease, the associated HPO annotations were acquired from [48], which covered disease-phenotype associations for over 6000 common, rare, infectious and Mendelian diseases through text-mining approach. The HPO-based disease similarities were defined by calculating the semantic similarity of their associated HPO phenotypes. For two diseases (*d*_*1*_, *d*_*2*_), the HPO-based similarity of *d*_*1*_ to *d*_*2*_ is defined as follows:

5 $$ HPO\_sim\left({d}_1\to {d}_2\right)=avg\left[{\displaystyle \sum_{s\in {d}_1}{ \max}_{t\in {d}_2} SemSim\left(s,t\right)}\right] $$

Where *s* and *t* are the annotated phenotypes of *d*_*1*_ and *d*_*2*_, respectively. *SemSim()* is one of the methods applied to compute the semantic similarity of two phenotype terms, including Resnik, Lin and Wang. Eq. 5, for each phenotype term of *d*_*1*_, found the “best match” among the phenotype terms annotated to *d*_*2*_, and the average overall phenotype terms was calculated. Note that this similarity is asymmetric, i.e., *HPO_sim*(*d*_*1*_ → *d*_*2*_) is not always equal to *HPO_sim*(*d*_*2*_ → *d*_*1*_). Therefore, we used a symmetric HPO-based similarity, which is defined in Eq. 6:

6 $$ HPO\_sim\left({d}_1,{d}_2\right)=\frac{1}{2}HPO\_sim\left({d}_1\to {d}_2\right)+\frac{1}{2}HPO\_sim\left({d}_2\to {d}_1\right) $$

The DO-based disease similarities were defined as the directly semantic similarity of two disease terms in DO, where the above mentioned three semantic-base methods (Resnik, Lin and Wang) were applied, too. NetSim was also compared with other function-based methods including BOG [19], PSB [20] and FunSim [25]. Parameters of the aforementioned methods were set to values used in the original paper.

### Constructing disease similarity network (DSN)

Disease terms from DO were used as nodes in the similarity network between diseases (DSN). We computed the pair-wise similarity for a total of 3,201 diseases (with both associated genes and literature information) by the proposed method MedNetSim. If the similarity of a disease pair was ranked in the top 0.5 %, an undirected weighted edge between the disease pair was drawn. The network was visualized with the force-directed layout algorithm of Cytoscape [49] and colored according to top-level DO categories.

## Results and discussion

### Utilizing the entire network benefits disease similarity computation

**Fig 4:**
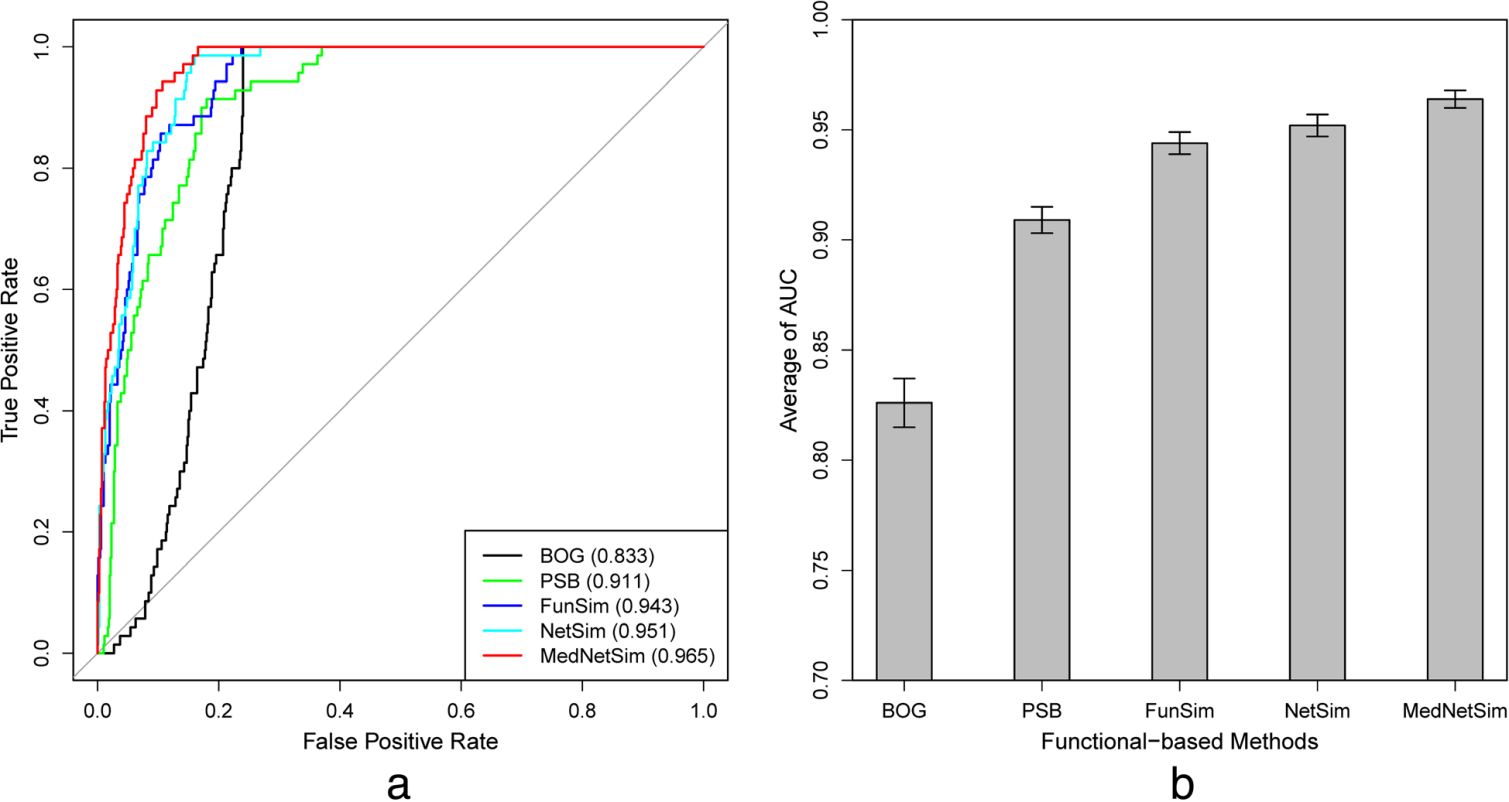
Performance of function-based methods. **a** ROC curves for the benchmark set and a random set. **b** Average AUC for the benchmark set and 50 random sets

Similarities of disease pairs in the benchmark set and a random set were calculated by NetSim and other function-based methods. As shown in Fig. 4a, the BOG method, with an AUC of 83.3 %, had the worst performance among function-based methods. Linking genes based on the GO biological process ontology [21], PSB method had significantly improved performance, achieving an AUC of 91.1 %. Considering nearest neighbors of disease-related genes in protein interaction network, FunSim improved its AUC to 94.3 %. The proposed method, NetSim, which utilized the entire protein interaction network, further improved its AUC to 95.1 %. The results show that utilizing the entire network can increase computing performance for disease similarity calculation. Integrating MedSim (see next section) and NetSim, the MedNetSim achieved the highest AUC among all function-based methods, improving its AUC to 96.5 %. The performance improvement indicates that integration of MEDLINE and protein interaction network can be an effective way to compute disease similarities. To check the stability of NetSim and MedNetSim, the above computation was repeated 50 times by calculating similarities using 50 randomly generated disease pair sets. Fig 4b shows the average AUC of BOG (82.6 %), PSB (90.9 %), FunSim (94.4 %), NetSim (95.2 %) and MedNetSim (96.4 %), which is consistent with Fig. 4a.

The MedNetSim similarity values of all disease pairs were computed, and a distribution of 5,121,600 similarity values (between 3,201 diseases) was acquired. The ranking of a similarity value in the distribution was used to compute its corresponding *p*-value. If the MedNetSim similarity value of a disease pair is in the highest-ranking 5 % of the distribution (which generates a *p*-value of 0.05), the two diseases are considered related. To evaluate the ability of MedNetSim in discriminating positive and negative cases, the *p*-values of similarities of disease pairs in the benchmark set and a random set were calculated (Additional file 4). For the benchmark set, 57 disease pairs were recognized as highly related diseases correctly and 13 disease pairs did not show a significant *p*-values (false negatives). The false negatives can be divided into two groups. The first group had a non-significant p-value of MedSim similarity, but a significant *p*-value of NetSim similarity, e.g., polycystic ovary syndrome (DOID: 11612) & myocardial infarction (DOID: 5844), malaria (DOID: 12365) & epilepsy syndrome (DOID: 1826) (Table 1). The missed calling of being positives for those disease pairs was mainly due to the very bad results of MedSim. That is to say, the research literature contains less information about their relatedness, therefore dragging down the performance of MedNetSim. For those disease pairs, NetSim may be a better choice. In the second group, both MedSim and NetSim similarities did not show significant *p*-values. A representative disease of the second group was lipid storage disease (DOID: 9455). 5 out of the 6 disease pairs between lipid storage disease and other diseases in the benchmark set were incorrectly identified, e.g., lipid storage disease & obesity (DOID: 9970), lipid storage disease & diabetes mellitus (DOID: 9351) (Table 1). The number of associated genes of obesity and diabetes mellitus was 1,527 and 1,134, respectively. Lipid storage disease only had 35 associated genes. Out of the 35 associated genes, 15 and 12 genes were shared by obesity and diabetes mellitus, respectively. Although more than 1/3 associated genes of lipid storage disease appeared in obesity and diabetes mellitus, they still got a bad NetSim results. That is because obesity and diabetes mellitus had a much bigger number of associated genes than lipid storage disease. This indicates that NetSim performs less well when two diseases have a large difference in the number of disease-associated genes. For the random set, 36 out of 700 disease pairs were recognized as related diseases (false positives). More than half of the 36 disease pairs were cancer related diseases, e.g., penile neoplasm (DOID: 11624) & cecum cancer (DOID: 1521), pancreatic cancer (DOID: 1793) & tubular adenocarcinoma (DOID: 4929) (Table 1). As cancer diseases were omitted in selecting benchmark set, it is not surprising that so many disease pairs related to cancers are detected as false positives. The relatedness of diseases belonging to different top-level DO categories was also identified, e.g., essential hypertension (DOID: 10825) & hyperthyroidism (DOID: 7998). Recently, Emokpae et al. had pointed out that hyperthyroidism was the most common thyroid disorder observed in patients with essential hypertension [50]. It indicates that our method can recognize related diseases which apparently seem unrelated. In addition, the relationship of impulse control disorder (DOID: 10937) & narcissistic personality disorder (DOID: 2745) was also detected (Table 1). The two disease are both in the “disease of mental health” (DOID: 150) category, but there is no report on their relatedness. Therefore, MedNetSim can also discover new unknown relatedness among diseases.

**Table 1:** Examples of false negatives and false positives with *p*-values from MedNetSim

Disease 1	Disease 2	*P*-value (MedSim)	*P*-value (NetSim)	*P*-value (MedNetSim)
False negatives				
polycystic ovary syndrome	myocardial infarction	0.663	0.004	0.051
lipid storage disease	obesity	0.107	0.148	0.070
malaria	epilepsy syndrome	0.675	0.016	0.075
lipid storage disease	diabetes mellitus	0.108	0.156	0.075
False positives				
impulse control disorder	narcissistic personality disorder	0.023	0.001	0.002
penile neoplasm	cecum cancer	0.023	0.007	0.004
pancreatic cancer	tubular adenocarcinoma	0.003	0.107	0.006
essential hypertension	hyperthyroidism	0.210	0.021	0.030

### MedSim can be a useful supplement to function-based methods

**Fig 5:**
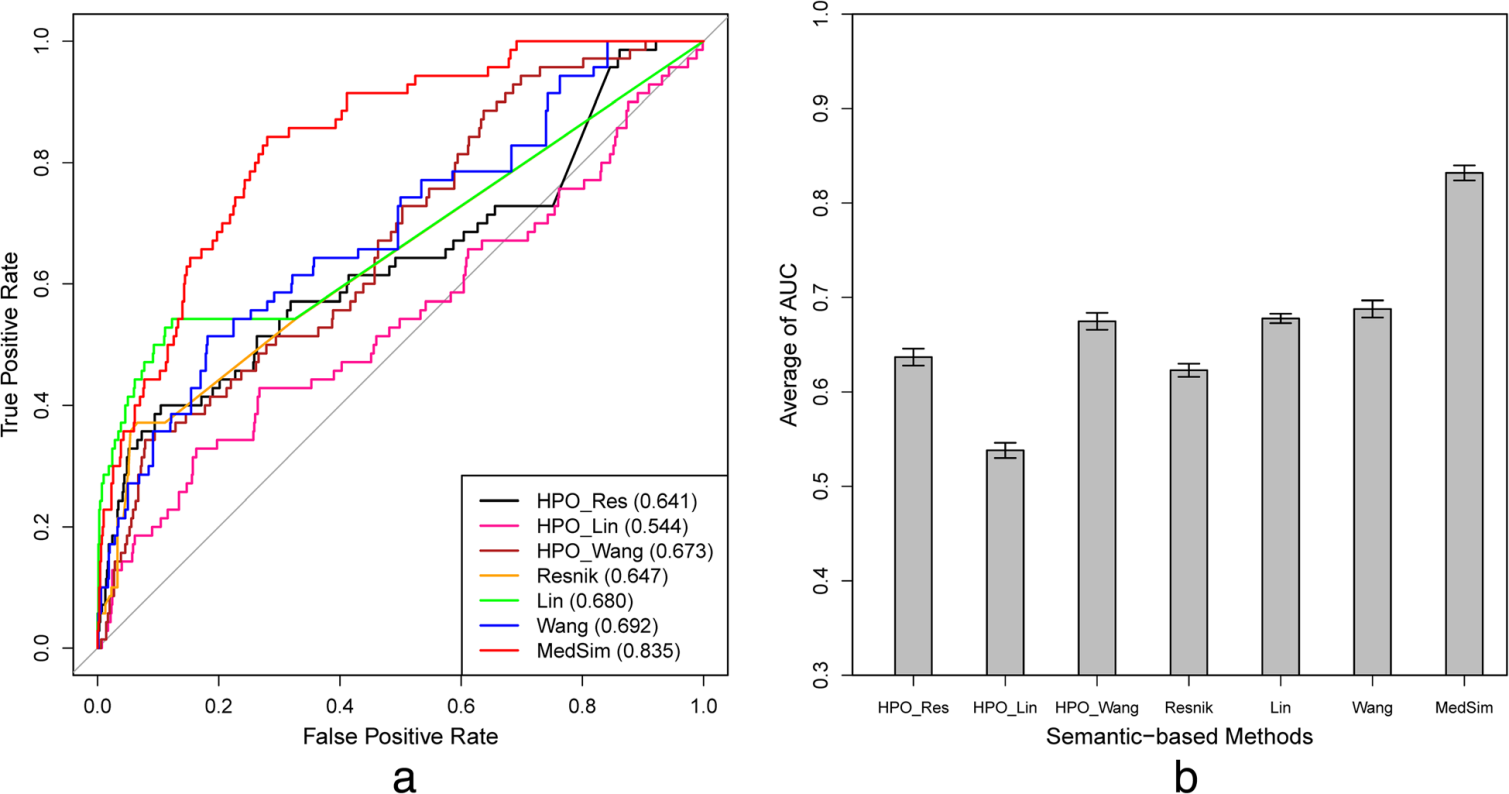
Performance of semantic-based methods. **a** ROC curves for the experimental results on the benchmark set and a random set. **b** Average AUC for the benchmark set and 50 random sets. HPO_Res, HPO_Lin and HPO_Wang denoted disease similarities computation by using Resnik, Lin and Wang based on HPO, respectively

ROC curves of MedSim and other semantic-based methods based on HPO and DO, respectively, were also generated (Fig. 5a). For the methods based on HPO, Lin’s method (HPO_Lin) had the worst performance with an AUC of only 54.4 %, and Wang et al.’s method (HPO_wang, 67.3 %) acquired the best performance among the three methods. As HPO was replaced by DO to calculate disease similarity, Resnik’s method (64.7 %) became the worst method, and Wang et al.’s method still had the best performance with an AUC of 69.2 %. Overall, performances of HPO-based methods are similar to DO-based methods. However, compared to computing disease similarity based on ontologies, the proposed MedSim had a significantly better performance than those methods. MedSim achieved an AUC of 83.5 %, which is even slightly better than the function-based method BOG. Figure 5b shows the average AUC for all semantic-based methods. The result is consistent with Fig. 5a.

Two reasons may explain why MedSim achieved the best performance among semantic-based methods. On the one hand, previous methods suffered from the incompleteness of ontologies and the lack of coverage of gene-disease or phenotype-disease association data. For example, only one-third of DO diseases have associated genes (see Additional file 1). HPO is widely used in the rare disease community [51]. However, the infrastructure of phenotype data for common and infectious diseases [48] is still developing. On the other hand, MedSim considered much richer and more diverse information included in literature, not only disease-related genes, but also disease symptoms, pathogenesis, therapeutic drugs, and so on.

MedSim requires only biomedical literature, no requirement to know disease-associated gene sets and ontologies. It thus has much broader applicability than previously published methods, especially in the case of no sufficient gene-disease association data.

### The impact of different data sources

#### Gene-disease association databases

**Fig 6:**
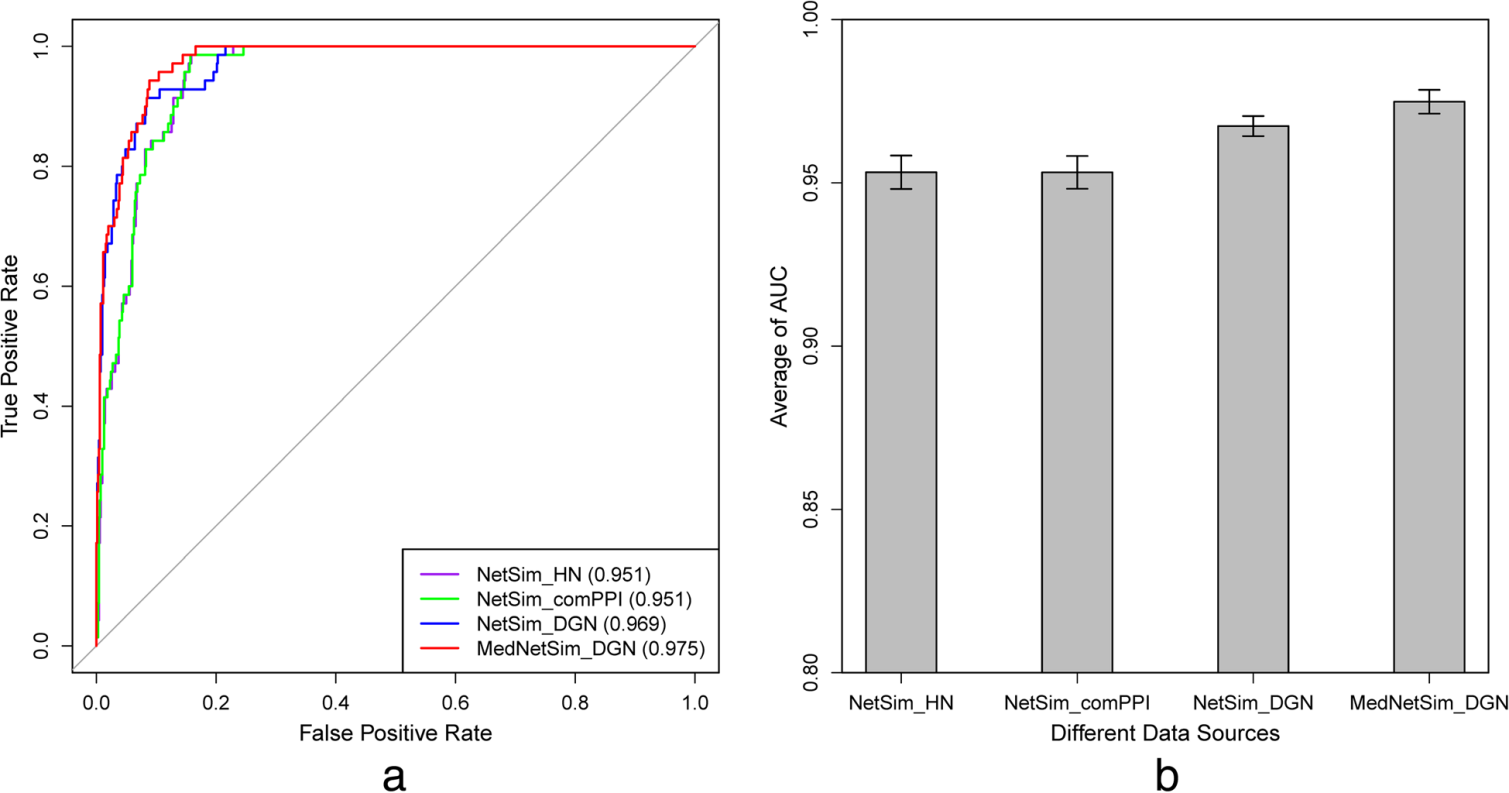
The impact of different data sources. **a** ROC curves for the experimental results on the benchmark set and a random set. **b** Average AUC for the benchmark set and 50 random sets

The effectiveness of different gene-disease association data was evaluated. DisGeNET was used as a replacement for SIDD. Compared to SIDD, DisGeNET has much more lower reliability associations based on literature mining. Its disease-gene associations are nearly two times of those in SIDD, with only 34 % more disease terms (Fig. 1). Using DisGeNET as gene-disease association data source, the AUC of NetSim (called as NetSim_DGN) grew to 96.9 % (Fig. 6a), which is even better than MedNetSim (AUC: 96.5 %, Fig. 4a) that fused MedSim and NetSim. Integration of MedSim and NetSim_DGN (MedNetSim_DGN) produced an AUC of 97.5 % (Fig. 6a). Fig. 6b shows the average AUC of NetSim_DGN (96.7 %) and MedNetSim_DGN (97.5 %), which is consistent with Fig. 6a too. The above observations show that a richer gene-disease association data, even with a lower reliability, is favorable for discovering relatedness between diseases.

#### Protein interaction datasets

To gauge the impact of different interaction datasets on computing performance, HumanNet database was used as the protein interaction network, substituting hPPIN. The number of protein nodes in HumanNet and hPPIN do not differ greatly, but the number of interactions in HumanNet is more than three times that of hPPIN (Fig. 2). However, the performance of NetSim while using HumanNet (named as NetSim_HN) did not improve at all compared to using hPPIN, with both achieving an AUC of 95.1 % (Fig. 6a). Furthermore, the common interaction pairs of hPPIN and HumanNet (i.e., comPPI) were also applied as the protein interaction network to evaluate the performance of NetSim (NetSim_comPPI, Fig. 6a). Although comPPI had a much smaller dataset than hPPIN or HumanNet, NetSim_comPPI achieved the same performance as NetSim and NetSim_HN, with an AUC of 95.1 % too. The average AUC of NetSim_HN and NetSim_comPPI (Fig. 6b) also showed the same results.

**Fig 7:**
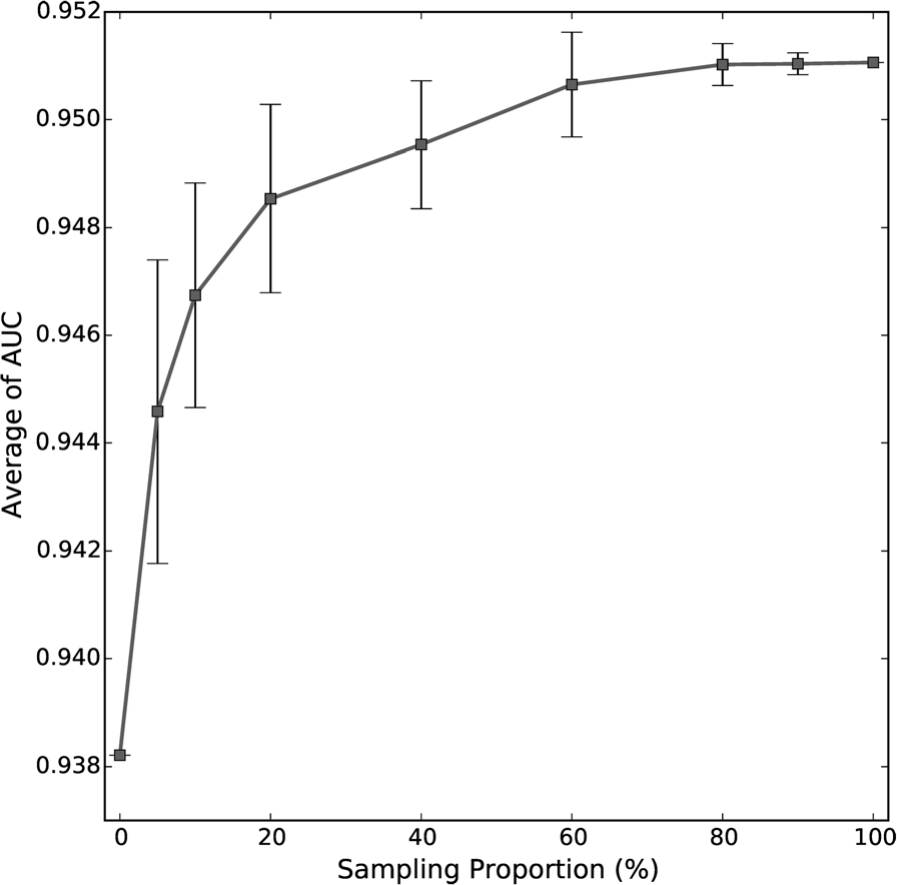
The average AUC of NetSim with different proportion of hPPIN sampled

Additionally, the average AUC of NetSim with different proportions of hPPIN were also computed. As shown in Fig. 7, the average AUC increased rapidly at the beginning, it then leveled off and did not grow as fast once the sampling rate hit 60 %. The average AUC plateaued at a sampling rate of 80 %. The above results indicate that merely using more protein interaction data does not lead to improved performance of NetSim. It might partially explain why using HumanNet, which has more than three times protein interaction data than hPPIN, did not improve the performance of NetSim.

Percentage of interaction pairs sharing GO annotation was analyzed for HumanNet, hPPIN and their common protein interactions (comPPI) (Table 2). For the entire GO annotation and its three categories (GO_BP: biological process, GO_CC: cellular component, GO_MF: molecular function), the percentage of pairs sharing annotation in hPPIN was higher than that in HumanNet, suggesting hPPIN has a higher data quality than HumanNet. The fact that HumanNet did not achieve improved performance for NetSim may partially be due to HumanNet’s lower data quality than that of hPPIN. In addition, whether the entire GO or its three categories, comPPI had the highest percentage of protein pairs sharing annotation in the three datasets, indicating that comPPI has the best data quality. The highest data quality of comPPI may be responsible for it acquiring same performance as that of hPPIN or HumanNet. All those results suggest that the quality of protein interaction data is more important than its volume for the computation of disease similarity.

**Table 2:** Percentage of interaction pairs sharing GO annotation

	GO	GO_BP	GO_CC	GO_MF
HumanNet	75.30 %	28.33 %	56.75 %	52.82 %
hPPIN	89.28 %	38.52 %	71.96 %	73.94 %
comPPI^a^	95.15 %	59.36 %	82.10 %	81.65 %

*GO* Gene Ontology, *GO_BP* biological process, *GO_CC* cellular component, *GO_MF* molecular function

^a^The common protein-protein interactions between HumanNet and hPPIN

### Disease similarity network

**Fig 8:**
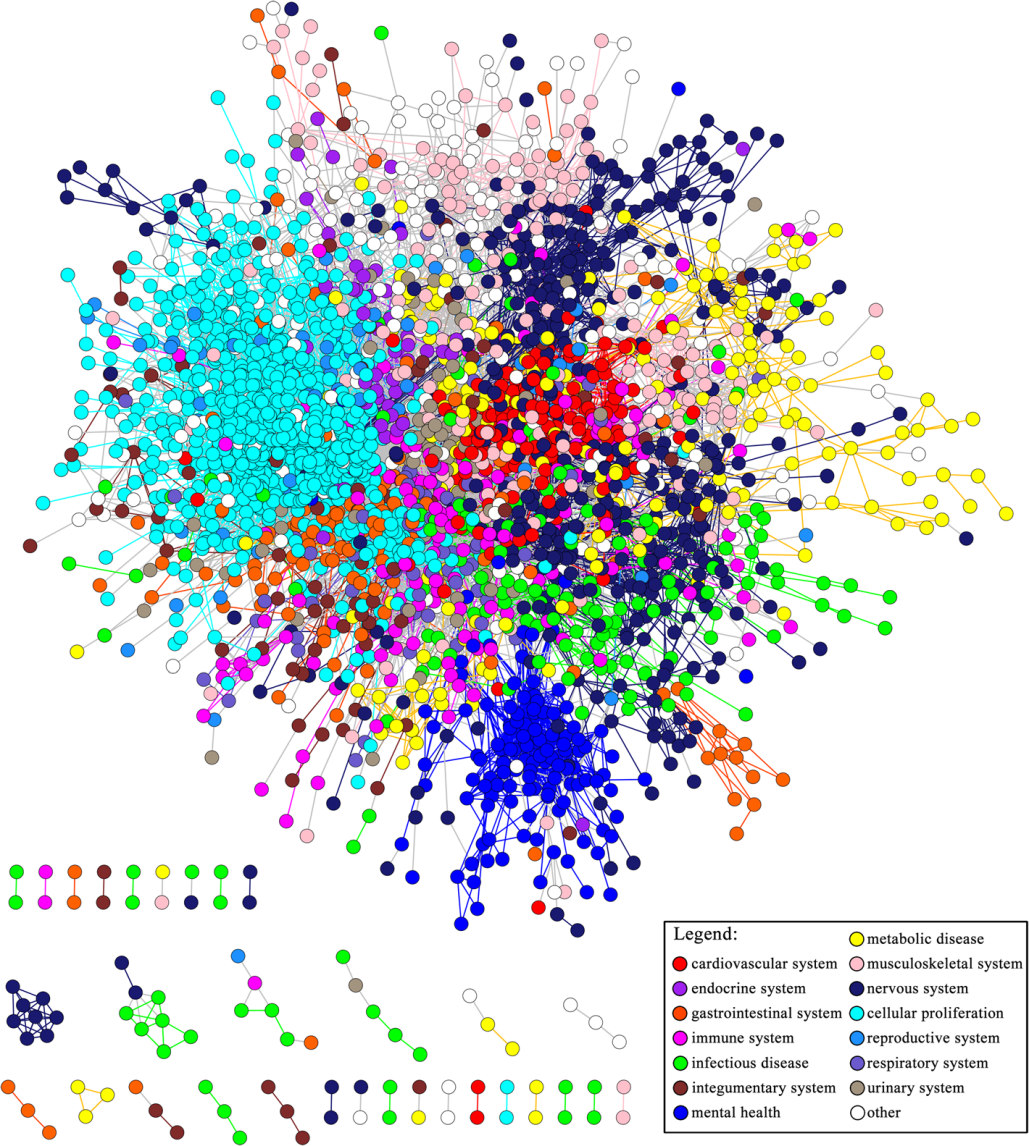
An overview of disease similarity network (DSN) based on MedNetSim results. The graph was based on a force-directed layout using the similarity between diseases as attraction force. Nodes were colored according to the top-level DO category to which they belong

As shown in Fig. 8, a disease similarity network (DSN) was generatedty based on MedNetSim from the top-ranking 0.5 % of pair-wise similarity values among 3,201 diseases in DO. 2,885 of the 3,201 diseases showed at least one connection to another disease, and 25,607 edges were formed between those diseases (Additional file 5). Each node in the network represented a disease. Those nodes belonged to 14 top-level DO categories and were colored according to their corresponding DO categories, such as “respiratory system disease” (DOID: 1579), “metabolic disease” (DOID: 0014667), “infectious disease” (DOID: 0050117), and so on. DO classified diseases both by anatomical site or system, and by general pathology. For each of the classifications, despite these different criteria, diseases within one category were usually in close proximity to each other (Fig. 8), such as “disease of cellular proliferation” (DOID: 14566), “disease of mental health” (DOID: 150), “nervous system disease” (DOID: 863), and so on.

**Fig 9:**
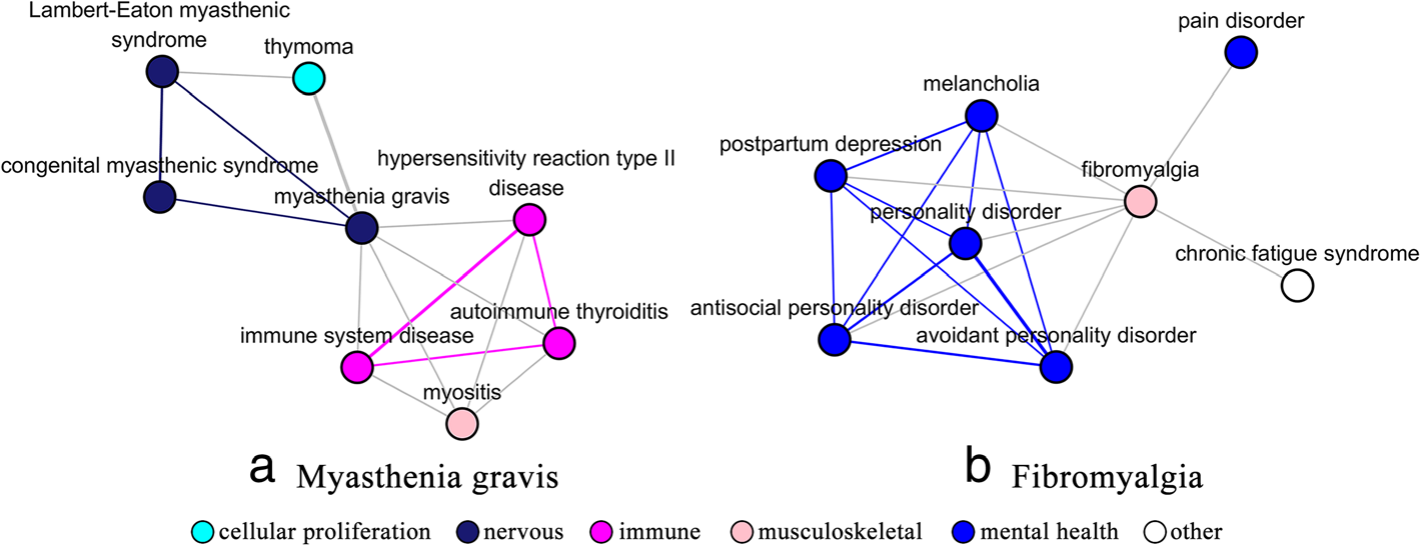
The sub-network around myasthenia gravis (**a**) and fibromyalgia (**b**). Nodes were colored according to membership in the top-level DO category. The thickness of the connections between the nodes reflects the degree of similarity

MedNetSim can also identify related disease groups belonging to different DO category. One example of these is myasthenia gravis (DOID: 437) which belongs to the “nervous system disease” (DOID: 863) category. Figure 9a showed the sub-network around myasthenia gravis (MG). It is not surprised that we found MG was related with “immune system disease” (DOID: 2914). Actually, MG is associated with various autoimmune diseases, including thyroid diseases [52] and lupus [53]. Thymoma (DOID: 3275) was found as the strongest associated partner of MG with a MedNetSim similarity up to 0.181 (*p*-value = 1.21 × 10^−4^), and vice versa. The relationship between thymic abnormalities and MG had also been reported [54]. Additionaly, MedNetSim can also be used to recognize new relatedness between diseases. Fibromyalgia (DOID: 631), belonging to the “musculoskeletal system disease” (DOID: 17) category, was taken as an example. As shown in Fig. 9b, fibromyalgia was associated to several mental health diseases, e.g., pain disorder (DOID: 0060164), postpartum depression (DOID: 9478). Studies has shown that fibromyalgia is frequently associated with depression and chronic pain [55]. There were a few reports on the relatedness between fibromyalgia and personality disorder (DOID: 1510) [56, 57]. However, fibromyalgia’s relationship with antisocial personality disorder (DOID: 10939) and avoidant personality disorder (DOID: 1509) are currently not reported. Interestingly, their associations were found in Fig. 9b. It was also found that melancholia (DOID: 2848) was related to fibromyalgia. Those new found relatedness between diseases might deserve further research to understand their common pathophysiology and help drug repositioning research.

## Conclusions

Methods based on protein interaction networks, literature data (MEDLINE), and their integration, were developed to compute disease similarity (NetSim, MedSim and MedNetSim). Taking advantage of the entire protein interaction network, NetSim obtained the best performance in all function-based methods. Among semantic-based methods, the performance of MedSim achieved significantly better results. MedSim does not require prior knowledge of disease-associated genes, enabling it to have a wider range of application than the other methods. MedSim can be a great supplement to function-based algorithms, especially when there is not enough gene-disease association data. The further improved AUC of MedNetSim shows that integrating biomedical literature and protein interaction data can be an effective way to improve computation for disease similarities.

Quality of protein interaction data was found to be more important than its volume, while higher volume of gene-disease association data, even with lower reliability, is more beneficial for disease similarity computation. In a situation of limited resources, it maybe beneficial to put more efforts toward obtaining more gene-disease association data and improving the quality of protein-protein interaction network.

MedSim, NetSim and MedNetSim are availalbe at http://www.digintelli.com:8000/. The user can enter two diseases of interest; the web service will compute their similarity and present a corresponding *p*-value.

## Additional files

Additional file 1: DiseaseGeneAssocSIDD.xls. Disease-gene associations acquired from SIDD database. (XLS 888 kb) Additional file 2: DiseaseGeneAssocDisGeNET.xls. Disease-gene associations acquired from DisGeNET database. (XLS 1582 kb) Additional file 3: Benchmark.xls. The benchmark set of related disease pairs. (XLS 25 kb) Additional file 4: SimilarityPvalues.xls. *P*-values of similarities of disease pairs in the benchmark set and a random set. (XLS 170 kb) Additional file 5: SimilarityNet.xls. The disease similarity network. (XLS 2552 kb)

## Abbreviations

AUC: The area under the ROC curve
BOG: Based on overlapping gene sets method
comPPI: Common protein-protein interactions of hPPIN and HumanNet
CTD: Comparative toxicogenomics database
DisGeNET: A database of gene-disease associations
DO: Disease ontology
DOID: Disease ontology identifier
DSN: Disease similarity network
GAD: Genetic association database
GO: Gene ontology
GO_BP: GO biological process
GO_CC: GO cellular component
GO_MF: GO molecular function
HPO: Human phenotype ontology
IC: Information content
IDF: Inverse document frequency
MeSH: Medical subject headings
MG: Myasthenia gravis
MICA: The most informative common ancestor
NLTK: Nature language toolkit
OMIM: Online mendelian inheritance in man
PSB: Process-similarity based method
ROC: Receiver operating characteristic
RWR: Random walk with restart
FR: Functional relevance
SIDD: A semantically integrated disease database
TF: Term frequency
TF-IDF: Term frequency times inverse document frequency
UMLS ID: Unified medical language system identifier
UMLS: Unified medical language system
